# Enhancement of Iron Acquisition in Rice by the Mugineic Acid Synthase Gene With Ferric Iron Reductase Gene and *OsIRO2* Confers Tolerance in Submerged and Nonsubmerged Calcareous Soils

**DOI:** 10.3389/fpls.2019.01179

**Published:** 2019-10-18

**Authors:** Hiroshi Masuda, May Sann Aung, Takanori Kobayashi, Tatsuro Hamada, Naoko K. Nishizawa

**Affiliations:** ^1^Research Institute for Bioresources and Biotechnology, Ishikawa Prefectural University, Ishikawa, Japan; ^2^Department of Biological Production, Faculty of Bioresource Sciences, Akita Prefectural University, Akita, Japan

**Keywords:** iron deficiency, transgenic rice, phytosiderophore, biomass, calcareous soil, *OsIRO2*, *IDS3*

## Abstract

Iron (Fe) is an essential micronutrient for plants. Plants encounter Fe deficiency when grown in calcareous soil with low Fe availability, leading to reduced crop yield and agricultural problem. Rice acquires Fe from the soil *via* Strategy I–related system (ferrous ion uptake by OsIRT1) and Strategy II system (ferric ion uptake by chelation). However, rice plants have a weak ability in Fe(III) reduction and phytosiderophore secretion. We previously produced an Fe deficiency–tolerant rice harboring *OsIRT1* promoter-*refre1/372* (for higher Fe(III) reductase ability) and a *35S* promoter-*OsIRO2* (for higher phytosiderophore secretion). In this study, we produced a new Fe deficiency–tolerant rice by the additional introduction of a barley *IDS3* genome fragment with *refre1/372* and *OsIRO2* (named as IRI lines) for further enhancement in Strategy II phytosiderophore productivity and better growth performance in various environments. Our results show that an enhanced tolerance was observed in *OsIRO2* introduced line at the early growth stage, *refre1/372* introduced line in the late stage, and RI line in all stages among five types of cultivation method. Moreover, we demonstrated that new IRI rice lines exhibited enhanced tolerance to Fe deficiency compared to nontransgenic (NT) rice and rice lines harboring the overexpressing *OsIRO2* or the *IDS3* fragment under submerged calcareous soil. The yields of IRI lines were ninefold higher than the NT line. Furthermore, under Fe-limited nonsubmerged calcareous soil condition (a new cultivation condition), IRI lines also conferred enhanced tolerance than NT, lines introducing only the *OsIRT1* promoter-*refre1/372* or overexpressing *OsIRO2*, and lines harboring both. Our results demonstrate that further enhancement of the Strategy II Fe uptake system by the mugineic acid synthase gene in addition to Fe uptake by enhanced ferric Fe reduction and phytosiderophore production in rice contributes Fe deficiency tolerance and broaden its utility in calcareous soil cultivation under paddy or nonpaddy field conditions.

## Introduction

Iron (Fe) is an essential micronutrient for the survival of most living organisms including plants. Approximately 30% of the Earth’s surface is covered by calcareous soil. There is an abundant amount of Fe in the soil. However, Fe is sparingly soluble under aerobic conditions, particularly in calcareous soil at high pH because Fe in soil mainly exists in its oxidized form (Fe^3+^) (Marschner, 1995). Plants often suffer from Fe deficiency when cultivated in calcareous soil and exhibit chlorosis symptoms primarily on young leaves, consequently affecting plant growth, crop yield, and quality. Therefore, producing Fe deficiency–tolerant crops will contribute to increasing crop production in widespread calcareous soils to fulfill the food demand for increasing world population.

Iron acquisition in higher plants is mediated by two major strategies: Strategy I and Strategy II (Römheld and Marschner, 1986). Under conditions of low Fe availability in soil, nongraminaceous species employ the Fe reduction system (Strategy I), in which Fe(III) in soil is first reduced to Fe^2+^ by Fe(III)-chelate reductase FRO2 in roots (Robinson et al., 1999), which is assumed to be the rate-limiting step of Fe uptake from soil (Connolly et al., 2003). Then, the resulting Fe^2+^ is transported across the plasma membrane of the roots by the Fe-regulated transporter, IRT1 (Eide et al., 1996). Rice (*Oryza sativa* L.) is a gramineous plant, but it also uses a part of the Strategy I system, which is a direct Fe^2+^ uptake system facilitated by the ferrous transporter OsIRT1 (Bughio et al., 2002; Ishimaru et al., 2006). Rice is typically cultivated in anaerobic paddy fields where abundant Fe^2+^ is readily available to plants because of the low redox potential. This is probably a reason why rice plants possess Fe^2+^ uptake system, although it is a gramineous plant (Bughio et al., 2002; Ishimaru et al., 2006). However, rice has limited Fe uptake efficiency due to its low Fe(III)-chelate reductase activity (Ishimaru et al., 2006) and also a lack of a complete Strategy I system.

All graminaceous species including rice employ Fe chelation system (Strategy II), in which plants synthesize and secrete mugineic acid family phytosiderophores (MAs) into the soil to acquire Fe. The biosynthetic pathway of MAs from methionine has been elucidated (Mori and Nishizawa, 1987; Shojima et al., 1990). In this pathway, nicotianamine (NA) is biosynthesized from three *S*-adenosyl-L-methionine by NA synthase (NAS) (Higuchi et al., 1999). Nicotianamine is synthesized into 2′-deoxymugineic acid (DMA) by NA aminotransferase (NAAT) (Takahashi et al., 1999; Inoue et al., 2008) and DMA synthase (DMAS) (Bashir et al., 2006). Rice plants synthesize DMA in root and secrete them to rhizospheres to chelate Fe(III), and the resulting Fe(III)–DMA complexes are transported into the plant roots *via* the Fe(III)-DMA transporter, OsYSL15 (Inoue et al., 2009; Lee et al., 2009). In many graminaceous species, DMA is further converted into various MAs by deoxygenases, such as IDS3 and IDS2 in barley (Nakanishi et al., 2000; Kobayashi et al., 2001). However, rice secretes only lower levels of MAs compared to other graminaceous species (Takagi, 1976; Marschner et al., 1986). This is a reason why rice is more susceptible to Fe deficiency than the other graminaceous plants.

Iron acquisition of the rice plant can be increased by transgenic approaches. In order to enhance MAs biosynthesis in the plant *via* Strategy II–based approach, first, Takahashi et al. (2001) introduced a barley genome fragment that contained the *HvNAAT-A* and *HvNAAT-B* genes into the rice, and the transgenic rice secreted higher amount of phytosiderophore than nontransgenic (NT) and exhibited tolerance to Fe deficiency in calcareous soil. In addition, Suzuki et al. (2008) developed transgenic rice lines carrying the barley genomic fragments containing genes (*HvNAS1*, or *HvNAS1* plus *HvNAAT-A* and -*B*, or *IDS3*), and all lines demonstrated tolerance to Fe deficiency when they were grown in a field with calcareous soil. Moreover, Masuda et al. (2013) also showed that combined introduction of the barley genomic fragments of MA synthesis genes (*HvNAS1*, *HvNAAT-A* and –*B*, and *IDS3*) into rice led to enhanced Fe deficiency tolerance and increased Fe concentration in grains. All these results suggested that improvement of phytosiderophore productivity in rice can obviously enhance Fe uptake and Fe deficiency tolerance in rice. The other Strategy II–based approach is the enhancement of Fe acquisition by overexpressing the transcription factor in rice. Ogo et al. (2006, 2007) identified an Fe deficiency–inducible basic helix-loop-helix transcription factor (OsIRO2), which mainly controls Strategy II–related Fe uptake key genes such as *OsNAS1*, *OsNAS2*, *OsNAAT1*, *OsDMAS1*, *TOM1*, and *OsYSL15*. When *OsIRO2* was introduced under the control of the constitutive cauliflower mosaic virus *35S* promoter into rice, the transformants had a larger amount of DMA secretion than NT plants and showed enhanced tolerance to Fe limitation in calcareous soil (Ogo et al., 2007; Ogo et al., 2011).

The Strategy I–based approach is the enhancement of Fe(III)-chelate reductase activity in the plant. Oki et al. (2004) artificially reconstructed and mutagenized to generate *refre1/372* from yeast Fe(III)-chelate reductase gene, *FRE1*. Refre1/372 shows increased enzymatic activity at alkaline pH, and it renders the transgenic tobacco plants resistant to Fe deficiency in calcareous soil (Oki et al., 2004). Ishimaru et al. (2007) introduced *refre1/372* into rice under the control of the *OsIRT1* promoter. The transgenic rice plants had enhanced Fe(III)-chelate reductase activity and conferred enhanced tolerance to Fe deficiency in calcareous soil (Ishimaru et al., 2007). Furthermore, these lines had a higher rate of Fe uptake and were 7.9-fold higher in grain yield than NT plants.

We recently developed an Fe deficiency–tolerant rice lines harboring *OsIRT1* promoter-*refre1/372* and overexpression of *OsIRO2* (Masuda et al., 2017). The introduction of a combination of two genes (*refre1/372* and *OsIRO2*; called “RI line”) was more effective to provide Fe deficiency tolerance in rice than the single introduction of either gene in early growth stage under water-submerged conditions (Masuda et al., 2017). However, in this cultivation condition alone, the advantage of the combination of *refre1/372* and *OsIRO2* was not clear at a late period of growth and yields compared to *refre1/372* alone. Moreover, the contribution of OsIRO2 alone may not be enough for the elevation of Fe deficiency tolerance at the late growth period. *IDS3* is a MAs biosynthesis gene, which is responsible for enhancing Fe uptake and translocation. Thus, further enhancement of Strategy II Fe uptake system by *IDS3* in rice together with *refre1/372* and *OsIRO2* is expected to be effective in enhancing Fe deficiency tolerance in calcareous soils.

In the present study, first, we observed the response of Refre1 line, OsIRO2 line, and RI lines to various cultivation conditions in calcareous soil and investigated the contribution of Fe deficiency tolerance by introducing *refre1/372*, *OsIRO2*, or combination of both genes. Then, we engineered a new construct and produced a rice line harboring an *IDS3* genome fragment, *OsIRT1* promoter-*refre1/372*, and *35S* promoter-*OsIRO2*. These transgenic lines possess *IDS3-refre1/372-OsIRO2* and are referred to as “IRI lines.” The growth and yield of rice are also highly influenced by other cultivation conditions such as water level and the type of fertilizers. Thus, we also observed the Fe deficiency tolerance and response of IRI lines compared to NT plants, the *OsIRO2* line, *Refre1* line, and RI line in calcareous soil under submerged and nonsubmerged conditions.

## Materials and Methods

### Production of *IDS3-Refre1/372-OsIRO2* (IRI) Rice Lines

The plasmid pIG121Hm containing a *35S* promoter-*OsIRO2*, *OsIRT1* promoter-*refre1/372*, and *IDS3* genome fragment was prepared as follows: the vector of RI line containing the *OsIRT1* promoter-refre1/372 and *35S* promoter-*OsIRO2* (Masuda et al., 2017) has a *Pme*I restriction enzyme site between RB and *35S-NPTII-tNOS* of the pIG121Hm vector (**Figure S1**). The *IDS3* genome fragment (Nakanishi et al., 2000) was digested by *Kpn*I and *Bam*HI, blunted by T4 DNA polymerase, and ligated to the RI vector, which was digested with *Pme*I. Then, the *IDS3*-*OsIRT1* promoter-*refre1/372-35S-OsIRO2*-pIG121Hm vector was obtained and named as IRI vector (**Figure S1**). Insertion of the *IDS3* genome fragment in the IRI vector was confirmed by polymerase chain reaction (PCR) with *IDS3* forward and reverse primers (5′-AAG CTT ACT GGT TGG ACG GTA TTT CA-3′ and 5′-GGA TCC ACG GGC CAC ATG ATC CA-3′, respectively), as well as by sequencing. *Agrobacterium tumefaciens* (strain C58) was used to introduce the IRI construct into the rice (*O. sativa* L. cv. Tsukinohikari) according to the modified method outlined in Hiei et al. (1994). Seventy-three regenerated plants were obtained. Gene insertion in regenerated plants was confirmed by genomic PCR. Then, IRI transformants were cultivated in a greenhouse at 28°C under natural light, and mature T_1_ seeds and also T_2_ seeds of the next generation were obtained for further analyses.

### RNA Extraction and Quantitative Real-Time Reverse Transcription–PCR Analyses

The rice seeds of NT and IRI transgenic rice (T_2_ seeds) were germinated for 17 days on Murashige and Skoog (1962) (MS) medium with and without hygromycin B (50 mg/L) for transformants and NT, respectively, at 28°C under 24-h light conditions. During the acclimation period of plantlets for 5 days, a leaf was sampled from each plantlet for the analyses of *OsIRO2* expression as described below. Then, IRI lines with higher expression of *OsIRO2* and NT plantlets were cultivated in an Fe-deficient hydroponic culture solution containing 122 mg/L K_2_SO_4_, 7.5 mg/L KCl, 14 mg/L KH_2_PO_4_, 472 mg/L Ca(NO_3_)_2_ • 4H_2_O, 123 mg/L MgSO_4_ • 7H_2_O, 0.62 mg/L H_3_BO_3_, 0.12 mg/L MnSO_4_ • 5H_2_O, 0.14 mg/L ZnSO_4_ • 7H_2_O, 0.05 mg/L CuSO_4_ • 5H_2_O, and 0.012 mg/L (NH_4_)_6_Mo_7_O_24_ as described in Masuda et al. (2017). The pH of the culture solution was adjusted to 5.5 every 2 days. After 6 days, roots were sampled for the analyses of *OsIRO2*, *refre1/372*, and *IDS3* gene expression. Total RNA was extracted from leaves or roots using an RNeasy Plant Mini Kit (Qiagen, Hilden, Germany). From extracted total RNA, the first-strand cDNA was synthesized using a ReverTra Ace qPCR reverse transcriptase (RT) Kit with gDNA remover (Toyobo, Osaka, Japan). Quantitative real-time RT-PCR was carried out using a StepOnePlus™ Real-Time PCR system (Applied Biosystems, Foster City, CA, USA) and SYBR Green (Takara, Shiga, Japan). The primers used for gene expression analyses were as follows: 5′-GGC ATG GCT CCC ATC GT-3′ and 5′-AAC AAG CTG ACC TGA ACC ATG A-3′ for *OsIRO2*, 5′-GGA AAG CTT CGT CTT CGC TTC-3′ and 5′-GAC CTC ATC ACG ACC AC TG-3′ for *IDS3*, and 5′-CCG AGA AGG TCT TCA GGA AC-3′ and 5′-CAT CCA TCC TAG TGT GTG GC-3′ for *refre1/372*. The plasmids that contain *HvNAS1*, *OsIRO2*, *IDS3*, or rice α-*tubulin* were diluted certain number of target gene copies (such as 10^3^, 10^4^, 10^5^, or 10^6^ copy/µl) and used as standard template. We performed real-time RT-PCR together with unknown cDNA template and standard templates, and then the copy number in unknown cDNA template was calculated based on standard line method. Transcript levels were normalized to the observed expression levels of α-*tubulin* by the primers 5′-TCT TCC ACC CTG AGC AGC TC-3′ and 5′-AAC CTT GGA GAC CAG TGC AG-3′, and the data is shown as “copy number of target gene/copy number of α-*tubulin*”. By agarose gel electrophoresis method, the sizes of the amplified fragments were confirmed.

### Growth Analyses of RI Lines on Calcareous Soil Under Different Water and Fertilizer Conditions

For the growth analyses in calcareous soil, T_3_ seeds of RI line No. 22 (Masuda et al., 2017), T_3_ seeds of Refre1 line No. 7 (Ishimaru et al., 2007), T_2_ seeds of IRO2 line No. 2 (Ogo et al., 2007, OX2), and NT seeds were used. Seeds were surface sterilized and germinated on MS medium with or without hygromycin B, at 28°C for 20 days. Then, after acclimation of germinated plantlets for 3 days, five plants of each line were transplanted to a pot containing 700 ml calcareous soil. This calcareous soil contains soluble CaO: 39.6% and Fe_2_O_3_: 1.7% with pH 8.9, and the source of the soil is Takaoka City, Toyama, Japan (Nihonkai Kougyou, Japan). To prevent pest contamination, the soil was autoclaved and used just before transplanting. Then, the plantlets were cultivated in the following five patterns: 1) Plants were supplied with the slow-release fertilizer Eco-long total 70 (ELT70; the fertilizer releases NPK and micronutrients within 70 days) containing nitrogen (N):phosphorus (P):potassium (K) (13%, 11%, 13%) with the micronutrients 0.20% Fe as EDTA-Na-Fe(III), 0.050% Cu, 0.015% Zn, and 0.020% Mo (JCAM AGRI, Co., Ltd., Tokyo, Japan) at a rate of 3 g/pot, and the water level of the container was maintained at lower than half of the pot height. 2) Plants were supplied with the slow-release fertilizer Eco-long 70 (EL70) containing N:P:K (14%, 12%, 14%) without micronutrients (JCAM AGRI, Co., Ltd.) at a rate of 3 g/pot, and the water level was maintained at lower than half of the pot height. 3) Hydroponic culture solution (50 ml) with Fe (components described above) was applied as fertilizer to each plant every 2 days, and the water level was maintained at lower than half of the pot height. 4) Hydroponic culture solution without Fe (components described above) was applied as a fertilizer into the whole cultivation box, and submerged conditions (water levels continuously higher than 2 cm of the pot height) were used. 5) ELT70 fertilizer was added at a rate of 3 g/pot, and the water level was maintained at higher than 3 cm above the pot height. A netting sheet was spread on the basal holes of the pot in order to avoid the roots from growing outside the pots. After setting up all conditions, the plants were cultivated in a greenhouse at 28°C under natural light. The SPAD value of the newest leaf and the shoot height were measured every 3 days by a SPAD-502 chlorophyll meter (Konica Minolta, Japan). Plants were harvested after seed maturation at 109 days after transplanting, and weights of panicles and straw were measured.

### Selection of IRI Lines on Calcareous Soil

Plantlets of IRI T_2_ lines with higher expression of *OsIRO2*, *Refre1/372*, and *IDS3*, as well as NT plantlets, were prepared as described above. Then, two plants of each line were transferred to a pot containing 700 ml calcareous soil as described above. A netting sheet was covered on the basal holes of the pot. Then, ELT40 (3.5 g/pot) and ELT70 (3.5g/pot) or hydroponic culture solution with or without Fe (50 ml/pot *per* week) was added as fertilizer. Submerged conditions (level of water continuously higher than 3 cm above the pot height) were used. The plants were cultivated in a greenhouse at 28°C under natural light. The shoot length and the SPAD value of the newest leaf were measured every 3 days throughout the cultivation. Then the lines with higher shoot length and higher SPAD value were selected for further analysis.

### Growth Analyses of IRI Lines on Calcareous Soils Under Submerged and Nonsubmerged Conditions

For the growth analyses of IRI lines on calcareous soil, T_3_ plantlets of IRI lines 1, 4, and 65; T_3_ plantlets of the RI line (Masuda et al., 2017; line No. 22); Refre1 line (Ishimaru et al., 2007; line No. 7); and IRO2 line (Ogo et al., 2007; OX2 line); T_5_ plantlets of the *IDS3* genome line (generated by Kobayashi et al., 2001 and tested the Fe deficiency tolerance in the field by Suzuki et al., 2008); and NT plantlets were prepared and cultivated in the following two patterns: 1) For the first experiment, individual plantlet was grown in each pot containing 1 kg of calcareous soil (as described above) with 3.5 g of ELT70 and 3.5 g of ELT140 fertilizers. A netting sheet was placed on the base of each pot. The growing method was the same as described in Masuda et al. (2017). The level of water was maintained at higher than 3 cm of the pot height. 2) For the second experiment, the same lines (T_3_ plants of IRI lines) were cultivated in a pot containing 1 kg calcareous soil, and 50 ml hydroponic culture solution without Fe was added as fertilizer to each pot every 3 days. Water was maintained at higher than 3 cm above the bottom of the cultivation box (one-fourth of the pot height). Shoot height and the SPAD value of the newest leaf were measured every 3 days. Weights of the plants were measured at 115 days after transplanting.

### Statistical Analyses

We used four biological replications (n = 4) for each variety for calcareous cultivation. Analysis of variance *via* Student's *t*-test was used to examine the experimental data from calcareous soil cultivation, such as plant height, SPAD value, and dry weights of panicles. Statistical analysis was performed using JMP14 software (SAS Institute, Cary, NC, USA); *p* < 0.05 was considered statistically significant.

## Results

### Production of Triple-Inserted IRI Lines and Selection of Lines With Higher Gene Expression Under Fe-Deficiency Conditions

**Figure 1:**
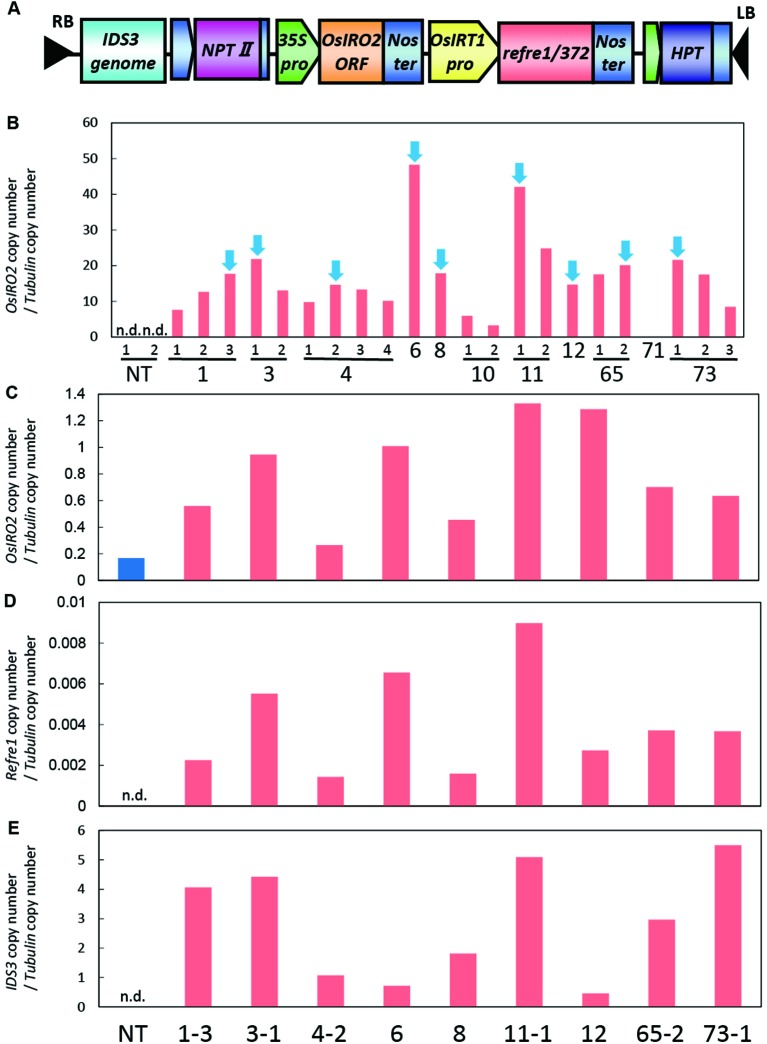
Vector construction and gene expression analyses of IRI rice lines. **(A)** Vector construction of IRI. This vector was introduced into rice cultivar Tsukinohikari by *Agrobacterium*-mediated rice transformation to produce IRI lines. **(B)** Gene expression analyses of *OsIRO2* by real-time RT-PCR for line selection. Leaves were sampled from T_2_ IRI lines and nontransgenic (NT) plants germinated on MS medium. The IRI lines with higher gene expression shown by blue arrows were selected for further analyses. **(C**–**E)** Expression of the three inserted genes, *OsIRO2*, *refre1/372*, and *IDS3*, respectively, in Fe deficiency roots by real-time RT-PCR. Roots were sampled from T_2_ IRI lines and NT plants cultivated in Fe-deficient hydroponic culture for 1 week. The expression levels were normalized with *rice* α-*tubulin*, and data shown represent target gene copy number/α-*tubulin* copy number (n = 1). n.d., not determined.

For the enhancement of Fe acquisition in rice, the IRI vector harboring the *IDS3*-*OsIRT1* promoter-*Refre1/372-35S-OsIRO2* was produced and introduced into rice plants by *Agrobacterium*-mediated transformation (**Figure 1A**, **Figure S1**). We obtained 73 regenerated lines, from which we selected the lines with higher expression levels of *OsIRO2* from the shoot of the T_2_ plants (**Figure 1B**). Next, the lines with higher expression of all three introduced genes (*OsIRO2*, *refre1/372*, and *IDS3*) were achieved by the real-time quantitative RT-PCR analysis of the roots of selected IRI lines grown in Fe-deficient hydroponic solution (**Figures 1C–E**). The expression levels of *OsIRO2* in the IRI lines were higher than those in the NT line (**Figure 1C**). The expression levels of *refre1/372* and *IDS3* were detected in IRI lines but not in the NT line (**Figures 1D, E**). Therefore, the nine T_2_ IRI lines with higher gene expression (lines 1-3, 3-1, 4-2, 6, 8, 11-1, 12, 65-2, and 73-1) were used for growth analyses and further investigation.

### Water Levels and Types of Fertilizers Influence Plant Growth of RI Lines

In order to determine the growth conditions that reflect the clear roles of individual gene and combined genes, first, Refre1 line, OsIRO2 line and RI line were grown on calcareous soil under five different cultivation conditions with varied fertilizer and water supply as follows.

**Cultivation condition (1):** When plants were grown with NPK fertilizer with micronutrients (ELT70) and under nonsubmerged conditions (**Figure S2A**), the growth of OsIRO2 line, Refre1 line, and RI line 22 was better than NT during vegetative stage (**Figure S2B**). However, all plants set panicles similarly during the maturation stage (**Figure S2D**), and there were no significant differences in the yields of all lines compared to NT lines (**Figure S2E**). The SPAD value was higher in RI line 22 compared to the OsIRO2, Refre1, and NT lines (**Figure S2C**).

**Cultivation condition (2):** When plants were grown with NPK fertilizer without micronutrients (EL70) and under nonsubmerged conditions (**Figure S3**), some NT plants died, but the transgenic lines survived (**Figure S3C**). The growth of the transgenic lines was better than the NT lines (**Figure S3A**). The SPAD values of the OsIRO2 and RI 22 lines were higher than NT and Refre1 lines in this condition (**Figure S3B**).

**Cultivation condition (3):** When plants were grown with an application of Fe-sufficient hydroponic solution under nonsubmerged conditions (**Figure S4A**), the growth, the SPAD values, and the yields of all transgenic lines were better than NT lines (**Figure S4**).

**Cultivation condition (4):** When plants were grown with application of hydroponic solution without Fe under submerged conditions (**Figure S5A**), the growth, SPAD values, and the yields of RI line 22 were better compared to the IRO2 and Refre1 lines, which were better than those of the NT lines (**Figures S5B–E**). In the early growth stage, the SPAD value of the OsIRO2 line was better than the Refre1 line (**Figures S5B, D**), and it exhibited the same trend as described in Masuda et al. (2017).

**Cultivation condition (5):** When plants were grown with NPK fertilizer with micronutrients (ELT70) and under submerged conditions (**Figure S6**), the RI line 22 and Refre1 grew better than the NT and OsIRO2 lines (**Figures S6A–D**).

We used nonsubmerged conditions for cultivation condition (1), (2), and (3) and submerged conditions for cultivation conditions (4) and (5). In this study, our results show that between the single introduction lines, Refre1 line was more tolerant than the IRO2 line under most cultivation conditions especially in the middle and late growth stages under cultivation conditions (1) and (5) (**Figures S2C**, **S6C**). On the other hand, the OsIRO2 line showed a better SPAD value especially in early growth stages, and the value decreased to the NT level in the middle and late growth stages under cultivation conditions (1) and (4) (**Figures S2C**, **S5D**).

### Fe Deficiency Tolerance of IRI Lines Under Various Cultivation Conditions

**Figure 2:**
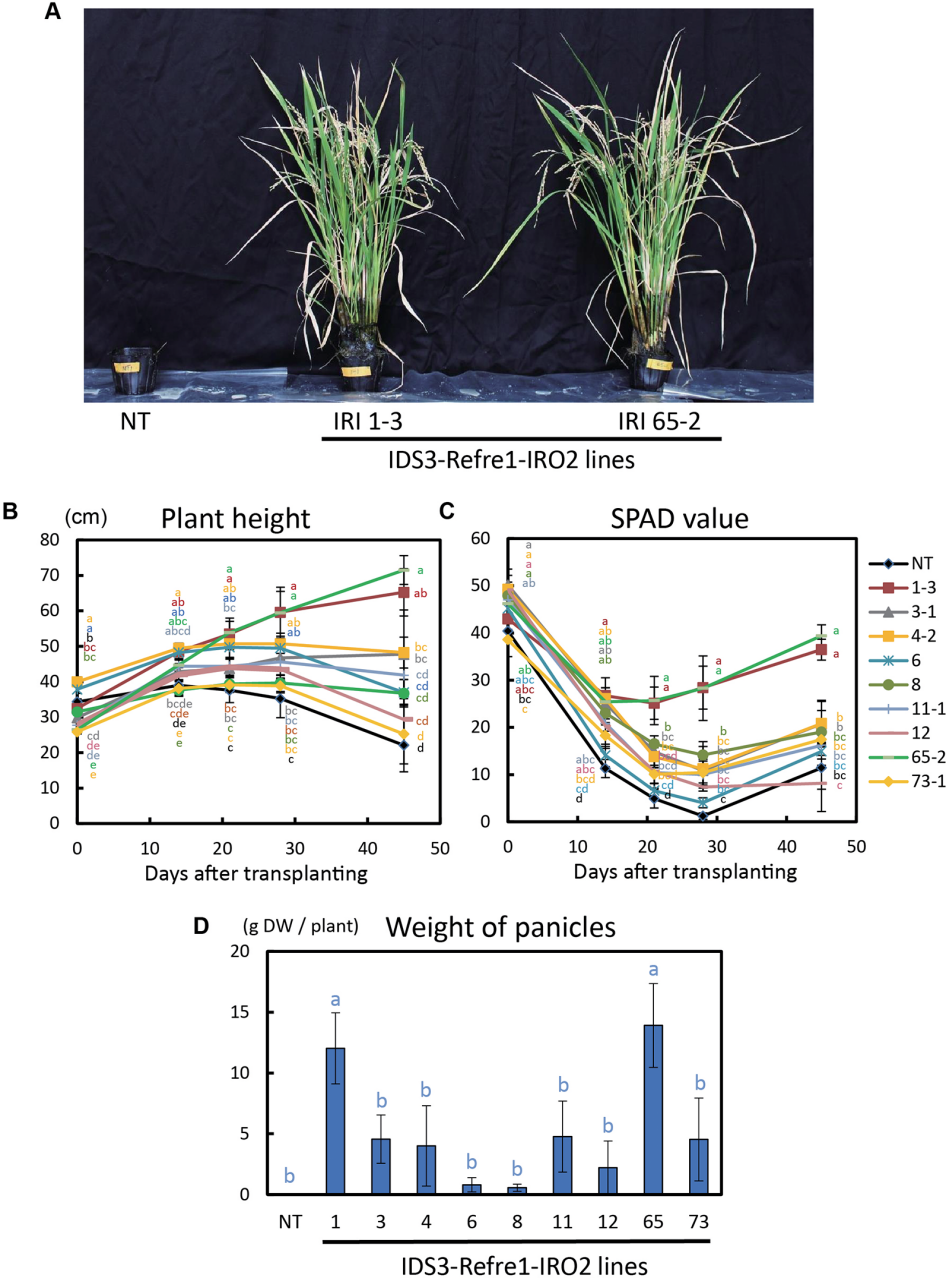
Growth test of IRI rice lines compared to NT on calcareous soil under water submerged condition. **(A)** Plant appearance at 117 days after transplanting. **(B)** Plant height. **(C)** SPAD value of the newest leaves. **(D)** Weight of panicles. NT, nontransgenic rice; Numbers, T_2_ plants of IRI lines. Error bars represent ± 1 standard error (SE) of biological replicates (n = 4). Values with different letters were significantly different by Student’s *t*-test (*p* < 0.05).

To further enhance Strategy II Fe uptake in rice, we focused on the introduction of the *IDS3*, a mugineic acid synthase gene of barley. We generated a new rice IRI line harboring the *IDS3* genome fragment, *OsIRT1* promoter-*refre1/372*, and *35S* promoter-*OsIRO2* (**Figure 1A**, **Figure S1A**). When nine T_2_ IRI lines with higher gene expression (lines 1-3, 3-1, 4-2, 6, 8, 11-1, 12, 65-2, and 73-1) were cultivated on calcareous soil with ELT70 fertilizer (NPK with micronutrients) under water-submerged conditions, the IRI rice plants exhibited superior Fe deficiency tolerance, while NT plants died (**Figure 2A**). The growth of IRI lines was better than that of NT lines (**Figure 2B**). In particular, the plant heights of lines 1-3, 3-1, 4-2, and 65-2 were better than the other lines. The SPAD values of lines 1-3 and 65-2 were higher than other lines, including NT lines (**Figure 2C**). The weights of panicles in lines 1-3 and 65-2 were also higher than other lines, including NT lines (**Figure 2D**). Thus, lines 1-3 and 65-2 were selected from this cultivation for further analyses.

**Figure 3:**
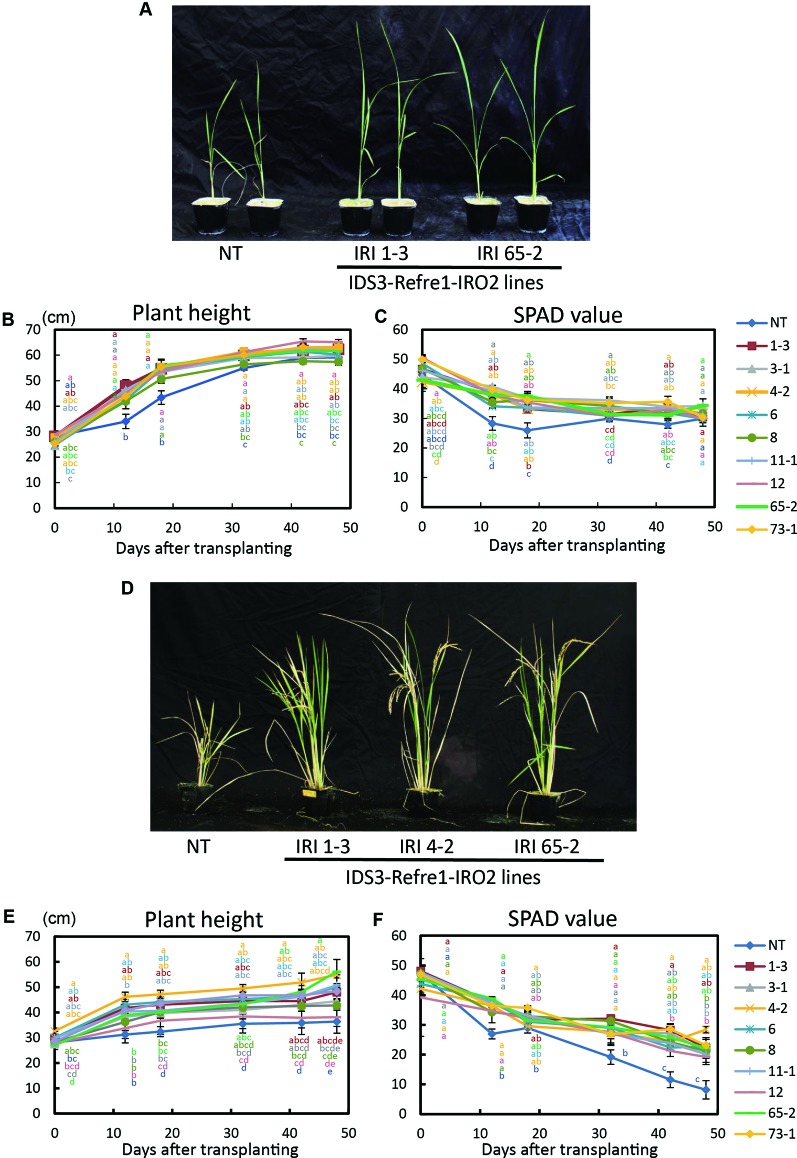
Growth test of IRI rice lines compared to NT on calcareous soil under non–water-submerged condition. **(A)** Plant appearance at 17 days after transplanting, **(B)** Plant height and **(C)** SPAD value of the newest leaves of the plants supplied by 50 ml Fe-sufficient hydroponic solution fertilizer to each pot every 3 days. **(D)** Plant appearance at 176 days after transplanting, **(E)** plant height, and **(F)** SPAD value of the newest leaves of the plants supplied by 50 ml Fe-deficient hydroponic solution fertilizer to each pot every 3 days. NT, nontransgenic rice; Numbers, T_2_ plants of IRI lines. Error bars represent ± 1 SE of biological replicates (n = 4). Values with different letters were significantly different by Student *t* test (*p* < 0.05).

Next, we grew the same lines in a calcareous soil under another cultivation condition, i.e., with Fe or without Fe hydroponic solution fertilizer under nonsubmerged conditions (**Figure 3**). With Fe-sufficient hydroponic solution fertilizer, although transgenic lines exhibited higher plant growth and SPAD values, a clear difference between each line and the NT lines was not apparent (**Figures 3A–C**). With the addition of Fe-deficient hydroponic solution, transgenic lines showed better growth and SPAD values compared to NT lines (**Figures 3D–F**). In particular, IRI line 4-2 showed better growth performance than other lines (**Figures 3E, F**). Thus, we also selected IRI line 4-2 for further analyses of calcareous soil cultivation.

### Growth of IRI Lines Under Fe-Sufficient Water-Submerged Conditions

**Figure 4:**
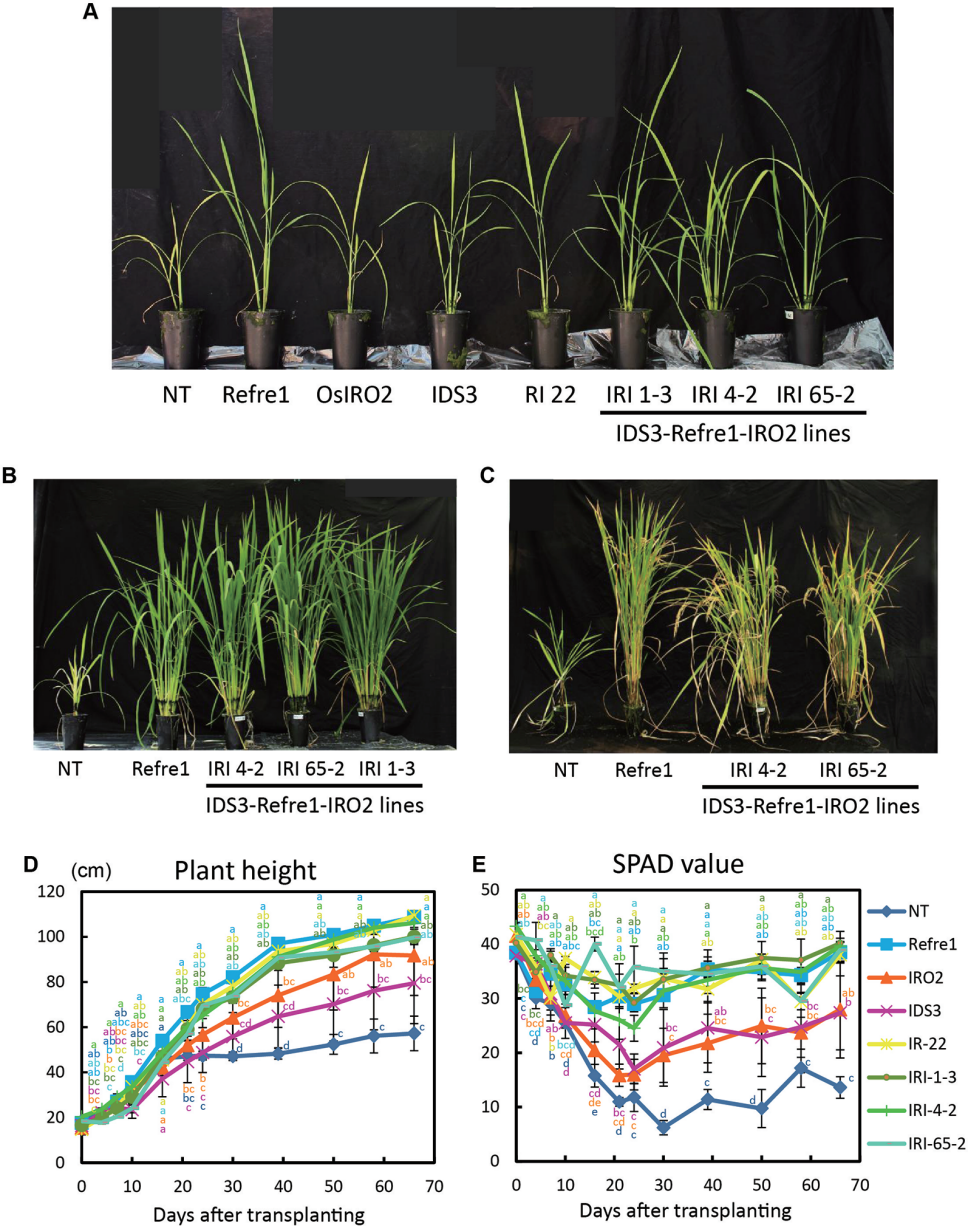
Growth test of IRI lines compared to NT and other lines on calcareous soil treated with Fe-sufficient fertilizer under water-submerged condition. **(A)** Plant appearances of all lines at 25 days after transplanting (DAT). **(B)** Plant appearances of IRI lines compared to NT and Refre1 lines during the vegetative stage at 73 DAT. **(C)** Plant appearances of IRI lines compared to NT and Refre1 lines during the maturation stage at 137 DAT. **(D)** Plant height. **(E)** SPAD value of the newest leaves. ELT fertilizer (NPK plus micronutrients) was applied to the soil. NT, nontransgenic rice; Refre1, line with *OsIRT1* promoter-*refre1*; IRO2, line with *35S* promoter-*OsIRO2*; IDS3, line with *IDS3* genome fragment; RI-22, line with *35S* promoter-*OsIRO2* and *OsIRT1* promoter-*refre1* No. 22; IRI, lines with *IDS3* genome fragment, *OsIRT1* promoter-*refre1*, and *35S* promoter-*OsIRO2*. Error bars represent ± 1 SE of biological replicates (n = 4). Values with different letters were significantly different by Student *t* test (*p* < 0.05).

To compare the contributions of individual genes introduced into IRI lines, we grew the previously produced IRO2 line (Ogo et al., 2007), Refre1 line (Ishimaru et al., 2007), IDS3 line (Masuda et al., 2008), and RI line No. 22 (Masuda et al., 2017) together with IRI and NT lines. When the plants were cultivated with ELT fertilizer (NPK plus micronutrients) under water-submerged condition (**Figure S7A**), IRI lines and all other transformants exhibited better growth than NT (**Figure 4A**). Especially, the IRI and Refre1 lines showed superior growth performance and Fe deficiency tolerance than the NT lines (**Figures 4B, C**). IRI lines 1-3, 4-2, and 65-2 and the Refre1 line showed better growth and SPAD values compared to the IDS3, IRO2, and NT lines (**Figures 4D, E**). The yields (panicle weight) and biomass (straw weight) of transgenic lines were higher than those of NT lines (**Table 1**, **Figure S8A**). The dry weights of straw were measured from the plants harvested at 150 days after transplanting for submerged condition (**Figure S8A**). After that long period, the other tested transgenic lines were also recovered likewise IRI and Refre1 lines especially under submerged condition (**Figure S8A**). However, there were remarkable differences in growth and SPAD among the lines (**Figures 4D, E**). Our results showed the Refre1 line was tolerant to Fe deficiency likewise IRI lines in this cultivation condition.

**Table 1:** Plant tolerance under calcareous soil cultivation with different water conditions.

Lines	Submerged condition - Calcareous soil		Nonsubmerged condition - Calcareous soil	
	Growth (Height, SPAD)	Biomass / Yield (g/plant)	Growth (Height, SPAD)	Biomass (g/plant)
NT	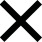 (57.3 ±7.7 cm, 13.6 ±2.0)	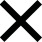 (15.5±3.6 /4.9±3.2)	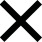 (71.1 ±0.7 cm, 13.75 ±1.6)	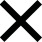 (3.1±0.2)
Refre1	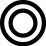 (108.8 ±2.9 cm, 38.5 ±1.8)	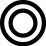 (90.7±14.2 / 9.6±6.9)	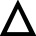 (76.5 ±1.5 cm, 20.2 ±4.9)	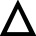 (3.9±0.3)
IRO2	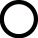 (91.8 ±17.7 cm, 28.0 ±8.9)	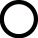 (54.5±21.3 /23.0± 9.7)	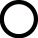 (79 ±2.0 cm, 22.9 ±1.8)	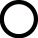 (5.0±0.4)
Refre1-IRO2 (RI line)	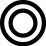 (109.4±1.1 cm, 38.0 ±1.5)	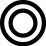 (53.9±5.0/26.0± 4.9)	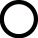 (75.8 ±1.8 cm, 25.0 ±6.8)	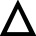 (4.1±0.6)
IDS3	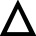 (79.5 ±17.9 cm, 27.4 ±6.8)	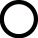 (36.9±17.2/14.0± 7.6)	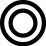 (79.6 ±3.3 cm, 36.1 ±2.1)	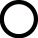 (5.3±0.2)
IDS3-Refre1-IRO2 (IRI line)	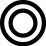 (106.1 ±1.9 cm, 39.7 ±1.4)	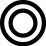 (50.0±10.3/21.4± 6.7)	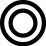 (81.8 ±1.6 cm, 36.9 ±2.4)	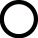 (5.2±0.4)

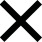
, very poor or weak; 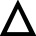, slightly good tolerance; 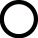, good tolerance; 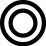, excellent tolerance. ± showed the standard error n = 4. The height of submerged condition, the SPAD of submerged condition, the height of nonsubmerged condition, and the SPAD of nonsubmerged conditions shown in the parentheses are referenced on 66 days after transplanting (DAT), 66 DAT, 33 DAT, 63 DAT, respectively. NT, nontransgenic rice. RI line represents RI line 22. IRI line represents IRI line 4-2.

### Growth of IRI Lines Under Fe-Limited Nonsubmerged Conditions

**Figure 5:**
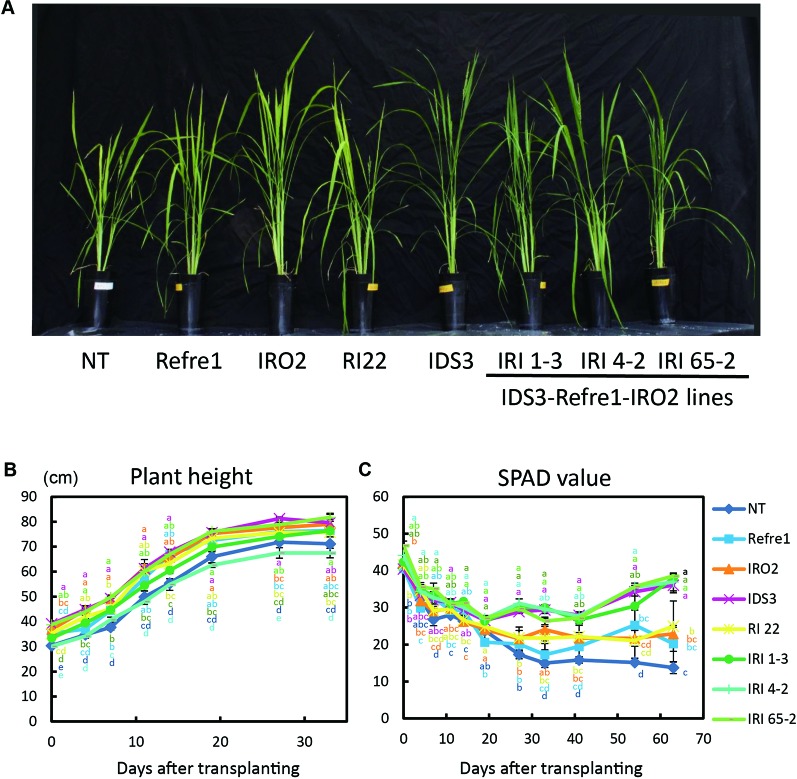
Growth test of IRI lines compared to NT and other lines on calcareous soil treated with Fe-deficient fertilizer under non–water-submerged condition. **(A)** Plant growth and appearances at 66 days after transplanting. **(B)** Plant height. **(C)** SPAD value of the newest leaves. Iron-deficient hydroponic solution fertilizer was applied to the soil. NT, nontransgenic rice; Refre1, line with *OsIRT1* promoter-*Refre1*; IRO2, line with *35S* promoter-*OsIRO2*; IDS3, line with *IDS3* genome fragment; RI-22, line with *35S* promoter-*OsIRO2* and *OsIRT1* promoter-*refre1* No. 22; IRI, lines with *IDS3* genome fragment, *OsIRT1* promoter-*refre1*, and *35S* promoter-*OsIRO2*. Error bars represent ± 1 SE of biological replicates (n = 4). Values with different letters were significantly different by Student *t* test (*p* < 0.05).

Transgenic and NT lines were cultivated on calcareous soil with Fe-deficient hydroponic solution fertilizer under nonsubmerged conditions (**Figure S7B**). In this cultivation condition, IDS3 and all IRI lines (line 1-3, 4-2, and 65-2) showed greenish leaves compared to NT, Refre1, IRO2, and RI22 lines, which showed clear Fe-deficiency chlorosis symptoms on leaves (**Figure 5A**). There was less difference in plant growth (**Figure 5B**). However, the SPAD values of IRI lines 1-3, 4-2, and 65-2 and IDS3 lines were higher than those of Refre1, IRO2, and RI line 22 (**Figure 5C**). The dry weights of straw were also measured from the plants harvested at 105 days after transplanting for nonsubmerged condition (**Figure S8B**). In this cultivation condition, the biomasses of the IRI lines, IDS line, and IRO2 line were better than those of Refre1 line, RI line 22, and NT (**Table 1**, **Figure S8B**).

## Discussion

### Water Levels and Fertilizer Types Influence Plant Growth of RI Lines

In our previous study (Masuda et al., 2017), the growth at late periods and yields between Refre1 and RI lines were similar, and the contribution of OsIRO2 to Fe deficiency tolerance was not clear. The plant growth is highly influenced by cultivation practices such as water levels and types of fertilizers, and thus the important roles of individual gene and combined genes on the stages of plant life would be interesting. In fact, the water level is not consistently maintained in real paddy field cultivation of rice. The Fe status in soil changes depending on the water level: Fe is mostly in precipitate form as ferric oxide under nonsubmerged condition, but it exists as a ferrous ion under submerged condition. Thus, the water level is an important factor in the cultivation of rice to resist Fe deficiency stress. Morikawa et al. (2004) used three types of fertilizer for rice cultivation (c.v. Tsukinohikari) in calcareous paddy fields: EL70, which includes only NPK (defined as CRF NPK in the study); ELT70, which includes NPK plus micronutrients (CRF M1 in the study); and ELT140, which also includes NPK plus micronutrients (CRF M2 in the study). All plants died when treated with EL fertilizer alone. The plants could not survive without micronutrient supplementation in the calcareous paddy field (Morikawa et al., 2004). It suggests that the type of fertilizer is another important factor for rice cultivation in calcareous soil.

Thus, we assumed that the determination of cultivation condition is important for Fe deficiency tolerance of plants grown on calcareous soil. In order to determine the growth conditions that reflect clear roles of individual gene and combined genes, we grew Refre1 line, OsIRO2 line, and RI line in five different cultivation conditions with varied fertilizers and water supplies with five different cultivation conditions (**Figures S2****–S6**). In all five cultivation conditions, the RI line showed the best growth. Our results suggested that, as concluded in Masuda et al. (2017), the combined introduction of *35S-IRO2* and *OsIRT1* promoter-*refre1* enhanced Fe deficiency tolerance in rice in calcareous soil than the single gene introduction.

In this study, our results show that among the single gene introduced lines, Refre1 line was more tolerant than the IRO2 line under most cultivation conditions except the early growth stage in cultivation condition (4) (**Figures S5B–D**). Under cultivation conditions (1) and (4), although the OsIRO2 line showed a better SPAD value in early growth stages, the value decreased to the NT level in the middle and late growth stages (**Figures S2C**, **S5D**). OsIRO2 plants may have grown well in the early growth stages because of increased expression of *OsIRO2*. However, in the middle and late growth stages, when plants grew larger and consequently the Fe demand of the plants increased further, the Fe uptake ability was not well enhanced by the introduction of *35S-OsIRO2* alone. We considered the further enhancement of the Strategy II Fe uptake system in rice would be required to produce higher Fe deficiency tolerance for calcareous soil cultivation.

### IRI Lines Exhibit Strong Fe Deficiency Tolerance Under Various Cultivation Conditions Compared to NT

We additionally introduced a barley mugineic acid synthase gene, *IDS3*, for the further enhancement of Strategy II Fe uptake in rice. Most of the rice variety including Tsukinohikari, the variety tested, can produce until only DMA, but it cannot produce mugineic acid (Kobayashi et al., 2001). When rice expresses *IDS3* by the introduction of the barley *IDS3* genome fragment, it can produce mugineic acid together with DMA, and the total amount of MAs secretion also increases (Kobayashi et al., 2001). Introduction of the *IDS3* genome fragment in rice enhances Fe translocation and increases seed Fe concentration (Masuda et al., 2008) and also enhances Fe deficiency tolerance in field cultivation on calcareous soil (Suzuki et al., 2008). We successfully generated IRI plants harboring the *IDS3* genome fragment, *OsIRT1* promoter-*refre1/372*, and *35S* promoter-*OsIRO2* (**Figure 1**, **Figure S1A**). IRI lines demonstrated excellent growth and superior Fe deficiency tolerance, while NT plants died when cultivated on calcareous soils with ELT fertilizer under submerged condition (**Figure 2**). Additional cultivation conditions with different fertilizer types were also used to determine the growth response on calcareous soils under nonsubmerged conditions (**Figure 3**). IRI lines exhibited higher growth and SPAD values in both fertilizer types (**Figure 3**), but there was no clear difference between each line and the NT lines with Fe-sufficient hydroponic fertilizer (**Figures 3A–C**), while a clear difference can be observed with Fe-deficient hydroponic fertilizer (**Figures 3D–F**). In this study, the two introduced genes, *OsIRT1* promoter-*refre1/372* and the *IDS3* genome fragment, were under the control of Fe deficiency–inducible promoters. Because of this fact, it may be indistinguishable that higher gene expression was as a result of the trait of particular lines or Fe deficiency. In our results, the gene expression of IRI lines 1-3, 4-2, and 65-2 was not the highest among the lines tested, but these lines showed remarkable Fe deficiency tolerance (**Figures 1B–E**), suggesting the need for line selection based on not only gene expression but also Fe deficiency tolerance.

### IRI Lines Showed Better Growth Under Fe-Sufficient Water-Submerged Conditions

To investigate the roles and contributions of individual genes introduced into IRI lines, all transformants were cultivated on calcareous soils with ELT fertilizer under water-submerged condition (**Figure S7A**). As a result, all transformants showed better growth than NT (**Figure 4A**). Especially, all IRI lines 1-3, 4-2, and 65-2 and the Refre1 line exhibited superior growth performance and Fe deficiency tolerance with high SPAD values compared to the IDS3, IRO2, and NT lines (**Figures 4B–E**). Our results showed the Refre1 line was tolerant to Fe deficiency likewise IRI lines (**Figure 4**). In fact, under anaerobic water-submerged condition, normal paddy soil has a large number of ferrous ions, especially in low pH soil. However, calcareous soil does not contain abundant soluble ferrous ions because of its high pH, and NT plants cannot take up Fe easily. Thus, under this water-submerged condition on high pH calcareous soil, *OsIRT1* promoter-*refre1/372* reduces ferric ions to ferrous form at the root surface, and ferrous form of Fe reduced by refre1/372 is easier to maintain in the reduction state of water-submerged cultivation and easier to take up directly by the OsIRT1 ferrous ion transporter in rice roots. This might be the main reason for the strong tolerance of the Refre1 line in this cultivation. *OsIRT1* promoter-refre1/372 showed high Fe(III)-chelate reductase activity and Fe deficiency–tolerant in calcareous soil (Ishimaru et al., 2007).

Another reason is partly that Refre1 line used MAs, which might be produced and released by the OsIRO2, IDS3, RI, and IRI lines under this cultivation condition. *OsIRO2*-overexpressing rice secreted DMA about 1.7 times (Ogo et al., 2007), and IDS3 rice secreted MAs about two times (Kobayashi et al., 2001) more than did NT plants. Because of the above reason, Refre1 was already stronger than NT under submerged condition. Therefore, these DMA and MAs produced by single, double, and triple introduced lines might be efficiently used by Refre1 line. In fact, the effect of MAs is lower, and MAs strategy is less effective under submerged condition. Thus, OsIRO2 and IDS3 lines were weak in this condition, and there was less difference between Refre1 and double introduced line. On the other hand, Refre1 rice had advantages in this condition.

As reported in Masuda et al. (2017), we confirmed *OsIRO2* overexpression improved Fe deficiency tolerance at the early growth stage of the plants under submerged conditions (**Figures S2C**, **S3B**) and nonsubmerged condition (**Figure S5D**). OsIRO2 is an important transcription factor that regulates the genes involved in Strategy II–related Fe uptake and translocation (Ogo et al., 2007; Ogo et al., 2011). Moreover, OsIRO2 positively regulates the expression of NA and DMA biosynthesis genes, and DMA and Fe(III)-DMA transporters under both Fe-deficient and -sufficient conditions, and *OsIRO2* overexpression confers increased DMA secretion under Fe-deficiency condition (Ogo et al., 2007; Ogo et al., 2011). Thus, it is thought that the IRO2 line is constantly ready for Strategy II–based tolerance to Fe deficiency before Fe deficiency arises, resulting in early growth tolerance in calcareous soil cultivation (Masuda et al., 2017). However, *refre1/372* was expressed under the control of the *OsIRT1* promoter, which induces gene expression mainly under Fe-deficient conditions in roots of the Refre1 line. Thus, the Refre1 line is prone to meet Fe deficiency before inducing tolerance (Masuda et al., 2017). As a result, there was a comparatively low SPAD value and weak tolerance of the Refre1 line to Fe-deficient conditions during the first week of growth in calcareous soils (**Figures 4E**, **5C**, **Figures S3B**, **S5D**).

During the middle and late stages of cultivation, IRI lines, RI lines, and the Refre1 line exhibited superior tolerance compared to the NT, IRO2, and IDS3 lines (**Figure 4**). The trends were the same as reported in Masuda et al. (2017). The Fe(III)-chelate reductase activity of Refre1 line was two times higher than that of NT line under Fe-deficient conditions (Masuda et al., 2017). This advantage might confer Refre1 line, and the lines possess *refre1/372* to be more resilient than the NT line during the middle and late growth stages of growth in calcareous soils. On the other hand, the IRO2 line and IDS3 line exhibited weak tolerance during these growth stages (**Figure 4**). MAs productivity was enhanced by the introduction of *OsIRO2* or *IDS3*. However, under water-sufficient condition, diffusion of MA phytosiderophores produced and released from roots might be increased under such condition, and the concentration of MAs in rhizosphere decreased. The dose-dependent DMA application to hydroponic culture solution affects the improvement of Fe nutrition (Araki et al., 2015). Therefore, improvement of the reduction strategy mediated by *refre1/372* would be highly effective in supporting Fe acquisition when Strategy II–based uptake is insufficient during the middle and late growth stages under water-submerged condition.

### IRI Lines With the Additional Introduction of an *IDS3* Genome Fragment Are Effective Under Fe-Limited Nonsubmerged Conditions

When transgenic and NT lines were cultivated on calcareous soil with Fe-deficient hydroponic fertilizer under nonsubmerged conditions, NT, Refre1, IRO2, and RI22 lines showed clear Fe-deficiency chlorosis symptoms on leaves, whereas IDS3 and all IRI lines exhibited greenish healthy leaves, and their SPAD values were higher than those of Refre1, IRO2, and RI line 22 (**Figures 5A, C**). Our results indicate that the Refre1 line did not tolerate under nonsubmerged condition (**Figure 5**). The reason for this could be that ferric ions which were reduced by *refre1/372* on root surface might be oxidized easily in the oxidation state under nonsubmerged conditions and difficult to take up by *OsIRT1* in rice roots.

OsIRO2 and RI lines also did not tolerate in this condition compared to NT. It might be because of two reasons. 1) The productivity of MA which normal rice cannot produce and secrete is important. NT, Refre1, OsIRO2, and RI plants can produce only until DMA. On the other hand, IDS3 line and IRI lines can produce both DMA and MA. MA is more efficient than DMA. 2) *OsIRO2* expression of the NT plant is very low under Fe-sufficient conditions compared to that of *35S*-promoter *OsIRO2* plant (Ogo et al., 2006), whereas it is dramatically high under Fe-deficiency conditions (Masuda et al., 2017). Thus, in middle and late growth stages of Fe-deficiency conditions in calcareous soils, this Fe deficiency–induced function of endogenous *OsIRO2* might outcome smaller contribution of *OsIRO2* overexpression in the IRO2 line than NT line and thus, the advantageous of *35S*-promoter *OsIRO2* gene is reduced compared to NT (Masuda et al., 2017, **Figures 4** and **5**). In this study, the contribution degree of the *35S*-OsIRO2 cassette when introduced together with *IDS3* genome and *refre1/372* is still unclear because the Fe deficiency tolerance of the line harboring refre1 and the *IDS3* genomic fragment has not been examined yet for comparison. Thus, in further study, the line harboring *Refre1-IDS3* cassette may be valuable as control compared with IRI lines to observe whether the *35S*-*OsIRO2* cassette is essential or not together with *IDS3* genome and *refre1/372* to achieve Fe deficiency tolerance.

Our results confirm that the contribution of OsIRO2 alone was insufficient for the elevation of Fe deficiency tolerance at the late growth period. On the other hand, MAs biosynthesis gene IDS3 complements this necessity. Under nonsubmerged conditions, phytosiderophore produced and secreted to root rhizosphere might not diffuse and effectively contributed to uptake Fe in IDS3 line and IRI lines. IDS3 line and IRI line maintained high SPAD value and showed remarkable tolerance under nonsubmerged condition (**Figures 5A, C**). We show that IRI rice with further improvement of Strategy II Fe uptake system mediated by *IDS3* obviously can enhance Fe deficiency tolerance in the rice.

### IRI Lines Confers Further Enhancement of Fe Acquisition and Tolerance in Rice in Both Submerged and Nonsubmerged Calcareous Soils

In the present study, we showed that IRI lines exhibit better growth performance and Fe deficiency tolerance than NT and other lines under various cultivation conditions. **Table 1** illustrates the toleration levels of these lines to Fe deficiency, and their biomass and yields. In this study, “single inserted line (Refre1)” shows tolerance under submerged condition (**Figure 4**) but less tolerance under nonsubmerged condition (**Figure 5**). “Single inserted line (OsIRO2)” shows tolerance under nonsubmerged condition (for the early stage) (**Figure 5**) but less tolerance under submerged condition (**Figure 4**). “Single inserted line (IDS3 genome)” shows tolerance under nonsubmerged condition (for the late stage) (**Figure 5**) but less tolerance under submerged condition (**Figure 4**). “Double inserted line (Refre1+OsIRO2)” shows tolerance under submerged condition (**Figure 4**) but less tolerance under nonsubmerged condition compared to a single inserted IDS3 line (**Figure 5**). “Triple inserted line (Refre1+OsIRO2+IDS3)” shows tolerance under both submerged condition (likewise Refre1) and nonsubmerged condition (likewise OsIRO2 and IDS3). In practical field cultivation, the water level is difficult to manage by farmers especially in developing countries with weak irrigation and drainage system. For example, Refre1 line may produce a high yield in the year of enough rainfall, however, it will be under risk of dying when less rainfall or drought period continues. IRO2 or IDS3 line is expected to survive under less rainfall period, however, it cannot produce enough yield in abundant rainfall season. On the other hand, new rice, IRI line, may grow healthy under less rainfall or drought condition and may also produce high yield under abundant rainfall condition. This is the superiority of the IRI rice, and it will be important for breeding and practical application. Our results confirm that IRI rice with the additional insertion of the *IDS3* genome fragment also contributes to improved Fe deficiency tolerance in rice under water-limited conditions. MAs, which are produced by IDS3, might further contribute to the uptake of precipitated ferric iron in a calcareous soil under water-limited cultivation.

## Conclusions

This study confirms that IRI rice lines with the *IDS3* genome fragment, *OsIRT1*promoter-*refre1/372*, and *35S-OsIRO2* exhibit increased Fe deficiency tolerance under various cultivation conditions on calcareous soil. The effects of these three genes in increasing Fe deficiency tolerance were additive, not synergistic. However, under real field cultivation, water supply and nutrient conditions will vary dramatically and are difficult to control by rice farmers in many cases. It is important to produce crops with Fe deficiency tolerance in both submerged and nonsubmerged conditions, and IRI rice has a superior performance of consistent Fe deficiency tolerance in various cultivation conditions; the presence of all three genes in rice improves Fe deficiency tolerance to a greater extent than the single or double introduction. Our results will help improve growth performance and grain yields of diverse rice cultivars grown in calcareous soil to meet the food demand by the increasing world population.

## Author Contributions

HM, MSA, and NN designed and coordinated the overall study. HM conducted all experiments, analyzed the data and wrote the manuscript, with assistance from MSA. TH provides some research facilities and technical suggestions. TK discussed the results and made suggestions. NN improved the manuscript.

## Funding

This research was supported by the Advanced Low Carbon Technology Research and Development Program from the Japan Science and Technology Agency (JST; Grant Number JPMJAL1107 to NN) and JST research fund from the Japan Ministry of Science and Culture (to NN).

## Conflict of Interest

The authors declare that the research was conducted in the absence of any commercial or financial relationships that could be construed as a potential conflict of interest.
