# Supplementary material for: Enhancement of Iron Acquisition in Rice by the Mugineic Acid Synthase Gene With Ferric Iron Reductase Gene and OsIRO2 Confers Tolerance in Submerged and Nonsubmerged Calcareous Soils
Source: Front Plant Sci. 2019 Oct 18;10:1179. doi: 10.3389/fpls.2019.01179 (PMC6813920; doi:10.3389/fpls.2019.01179)
Supplement: Supplementary file 1 [file DataSheet_1.pdf]

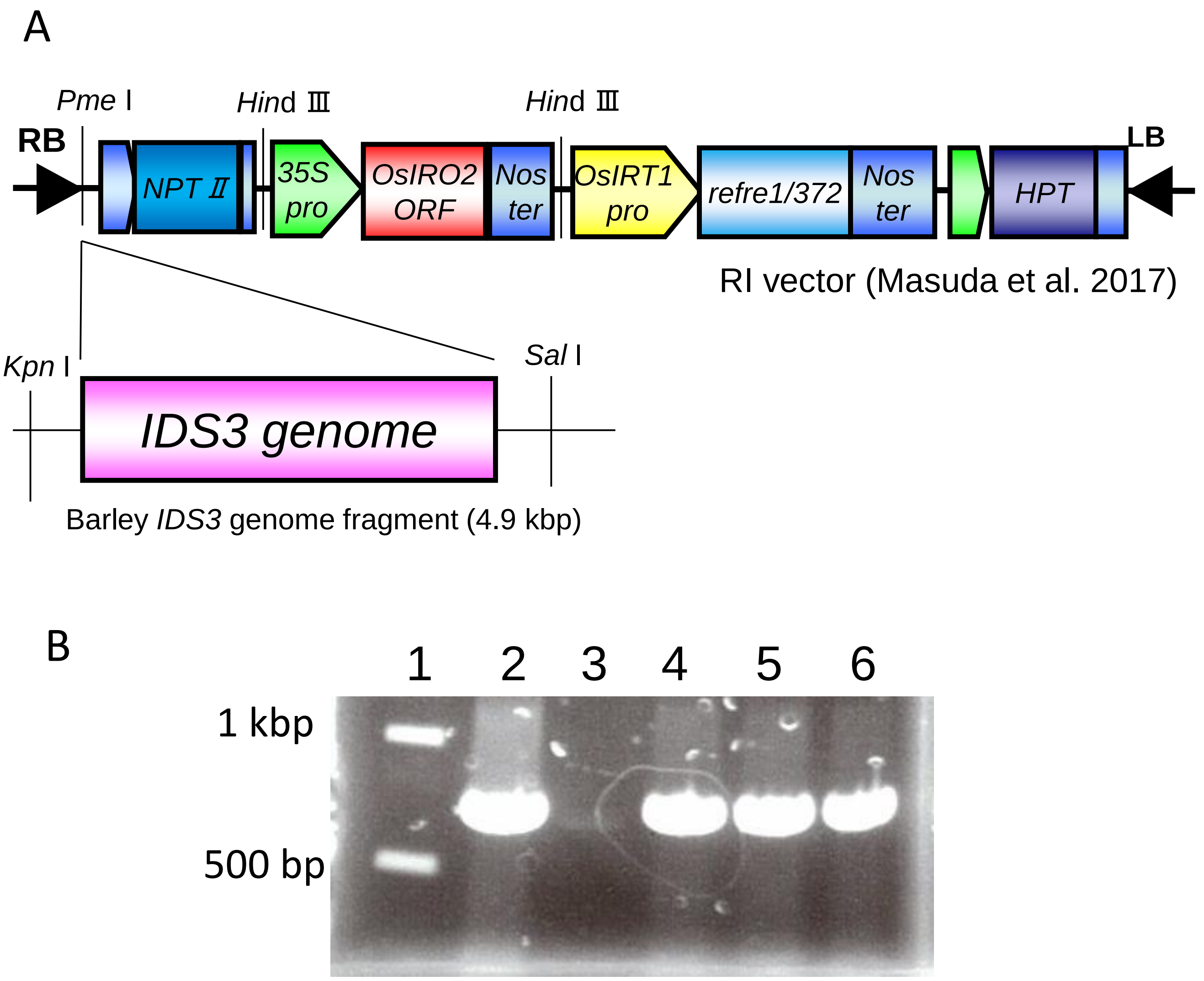

Supplementary Figure 1 | Construction of IRI vector. (A) Insertion site of *IDS3* genome in the RI vector. To produce IRI vector, *IDS3* genome fragment was digested by *Kpn* I and *Sal* I, blunted by T4DNA polymerase, then introduced to *Pme* I site of RI vector. (B) Confirmation of the insertion of *IDS3* genome fragment in IRI vector by PCR. 1: DNA Marker, 2: *IDS3* genome fragment of *IDS3* genome-SK vector, 3: RI vector, 4,5,6: IRI vector was used as DNA templates.

Supplementary Figure 1

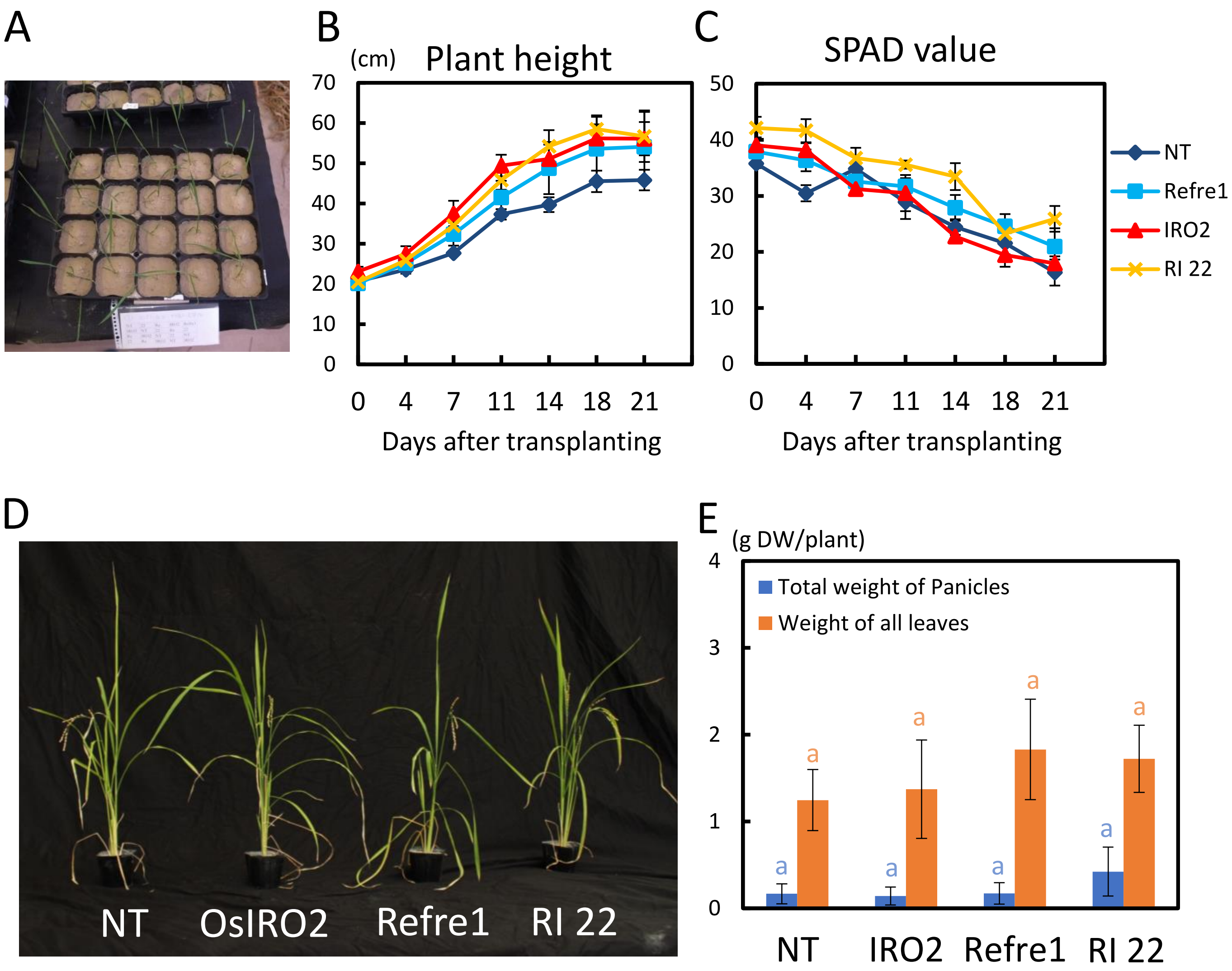

Supplementary Figure 2 | Cultivation condition 1: Growth test of RI rice on calcareous soil treated with ELT fertilizer (NPK plus micronutrients) under non-submerged conditions. (A) Cultivation condition at planting day. (B) Plant height. (C) SPAD value of the newest leaves. (D) Plant appearance at 83 days after transplanting. (E) Dry weights of panicles and shoot after harvest. Five plants of each line were planted in different pots and data is the average of 5 plants (n=5). NT: non- transgenic lines, Refre1: the line with *OsIRT1* promoter-*refre1/372*, OsIRO2: the line with *35S* promoter-*OsIRO2*, RI 22: RI line number 22 with *OsIRT1* promoter-*refre1/372* and *35S* promoter-*OsIRO2.* Error bars represent ± 1 SE of biological replicates, n = 5.

Supplementary Figure 2

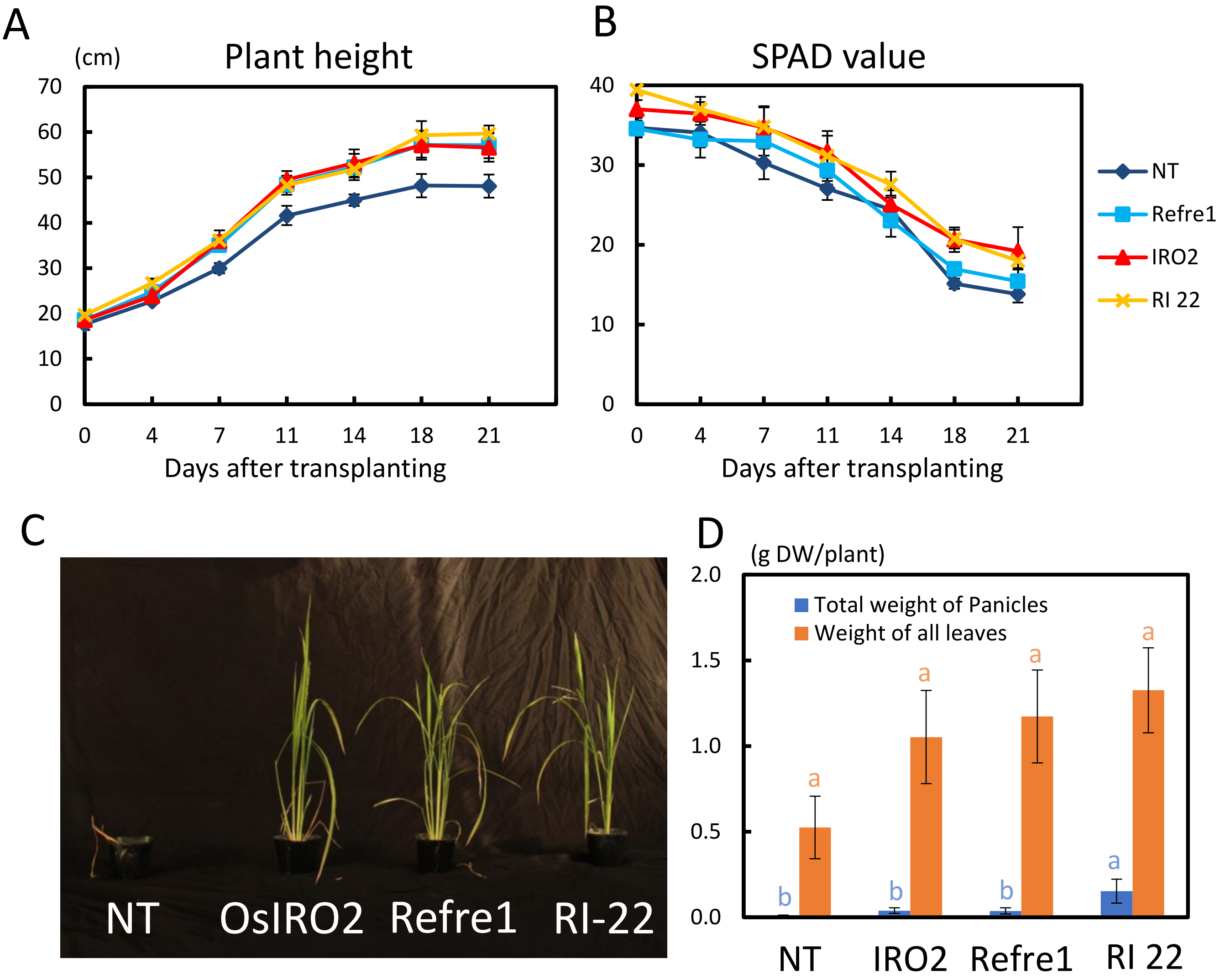

Supplementary Figure 3 | Cultivation condition 2: Growth test of RI rice on calcareous soil treated with EL fertilizer (only NPK) under non-submerged condition. (A) Plant height. (B) SPAD value of the newest leaves. (C) Plant appearance at 83 days after transplanting. (D) Dry weights of panicles and shoot after harvest. Five plants of each line were planted in different pots and data is the average of 5 plants (n=5). NT: non-transgenic lines, Refre1: the line with *OsIRT1* promoter-*refre1/372*, OsIRO2: the line with *35S* promoter-*OsIRO2*, RI-22: RI line number 22 with *OsIRT1* promoter-*refre1/372* and *35S* promoter-*OsIRO2.* Error bars represent ± 1 SE of biological replicates, n = 5.

Supplementary Figure 3

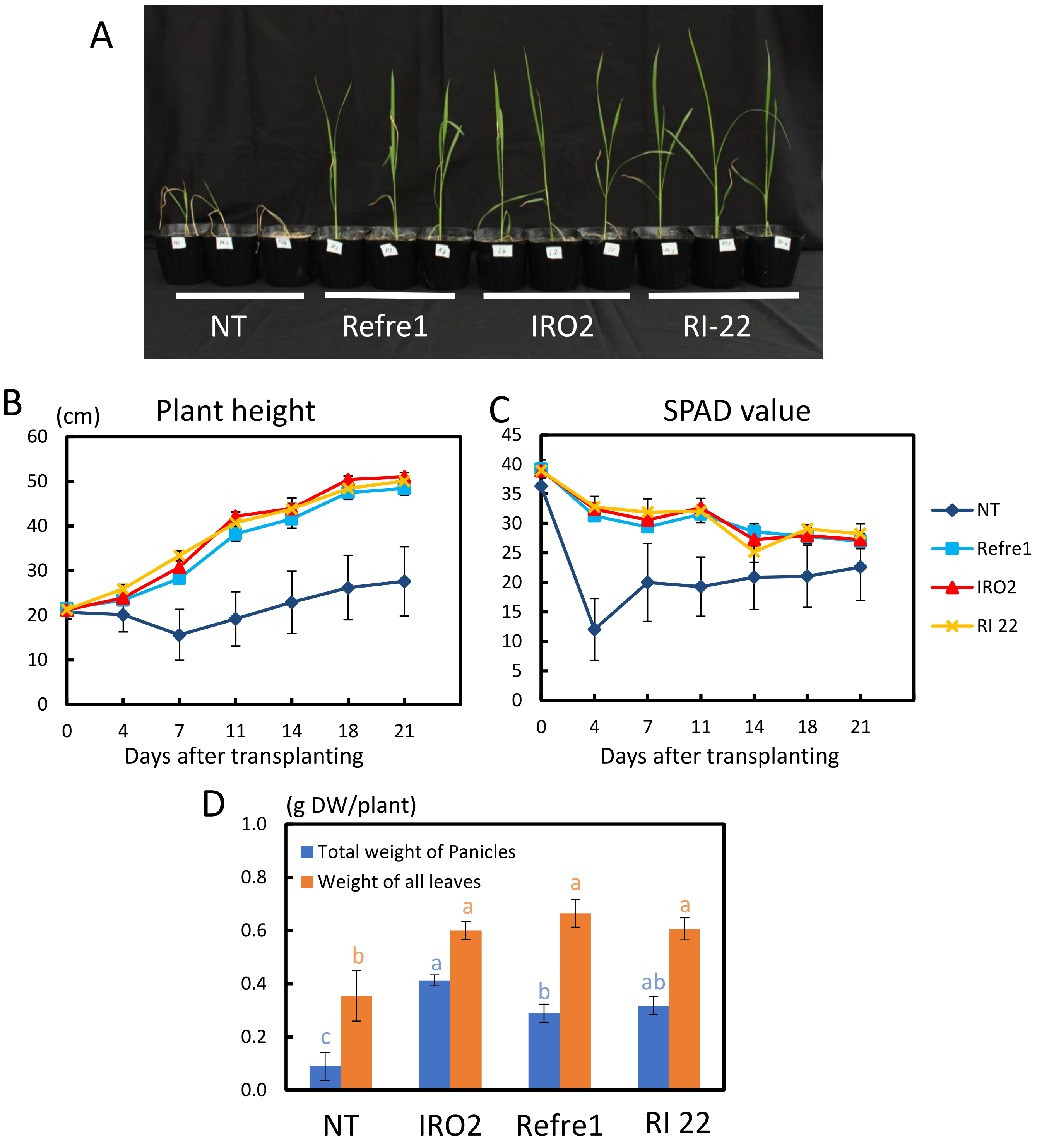

Supplementary Figure 4 | Cultivation condition 3: Growth test of RI rice on calcareous soil treated with Fe-sufficient hydroponic solution under non-submerged condition. (A) Plant appearance at 7 days after transplanting. (B) Plant height. (C) SPAD value of the newest leaves. (D) Dry weights of panicles and shoot after harvest. Five plants of each line were planted in different pots and data is the average of 5 plants (n=5). Hydroponic culture solution was added every two days as fertilizer. NT: non- transgenic lines, Refre1: the line with *OsIRT1* promoter-*refre1/372*, OsIRO2: the line with *35S* promoter-*OsIRO2*, RI-22: RI line number 22 with *OsIRT1* promoter-*refre1/372* and *35S* promoter-*OsIRO2.* Error bars represent ± 1 SE of biological replicates, n = 5.

Supplementary Figure 4

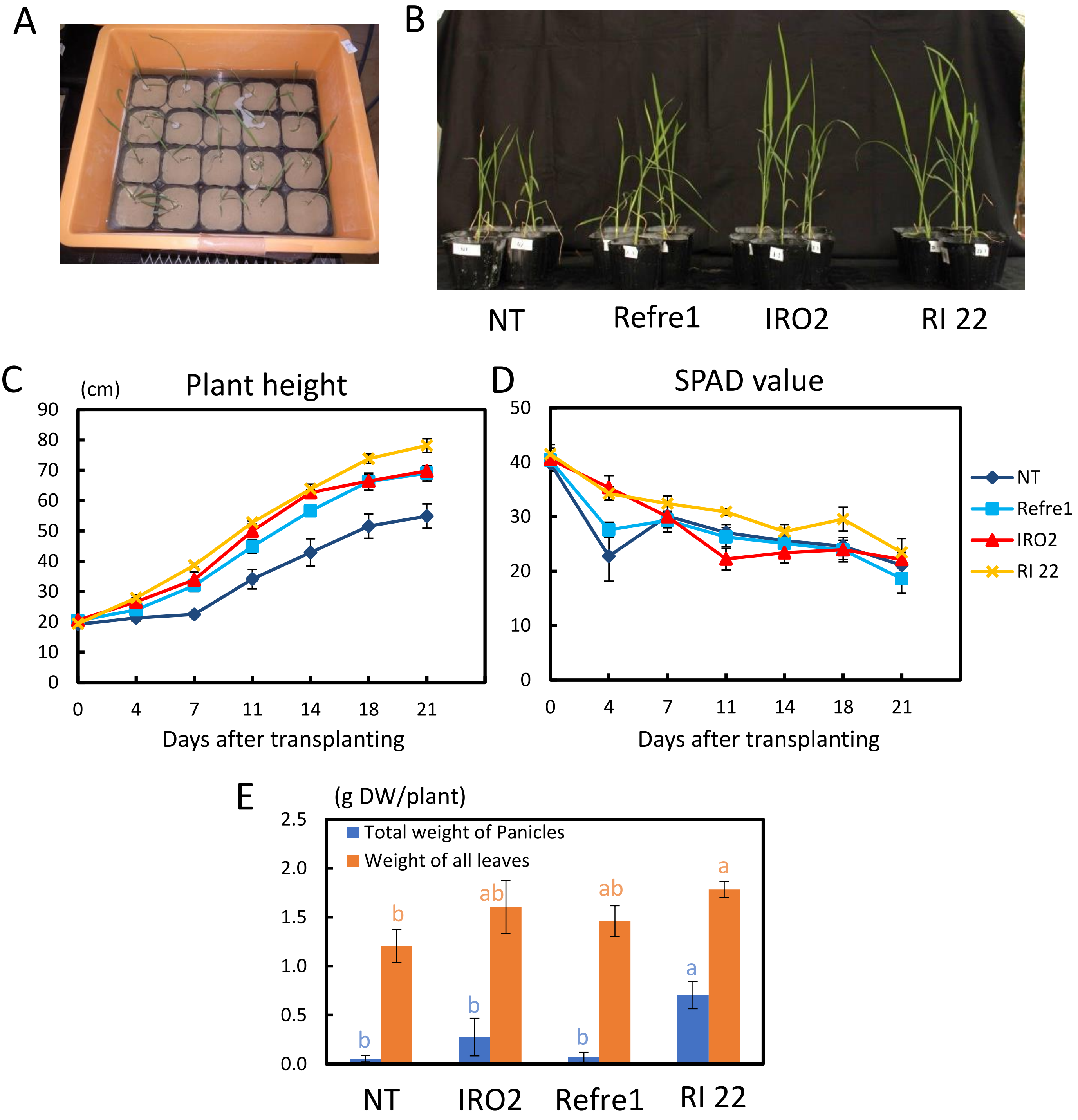

Supplementary Figure 5 | Cultivation condition 4: Growth test of RI rice on calcareous soil treated with Fe-deficient hydroponic solution under submerged condition. (A) Cultivation condition at planting day. (B) Plant appearance at 7 days after transplanting. (C) Plant height. (D) SPAD value of the newest leaves. (E) Dry weights of panicles and shoot after harvest. Five plants of each line were planted in different pots and data is the average of 5 plants (n=5). Hydroponic solution was added as fertilizer and water level was continuously maintained over 2 cm of the pot height. NT: non- transgenic lines, Refre1: the line with *OsIRT1* promoter-*refre1/372*, OsIRO2: the line with *35S* promoter-*OsIRO2*, RI-22: RI line number 22 with *OsIRT1* promoter-*refre1/372* and *35S* promoter-*OsIRO2*. Error bars represent ± 1 SE of biological replicates, n = 5.

Supplementary Figure 5

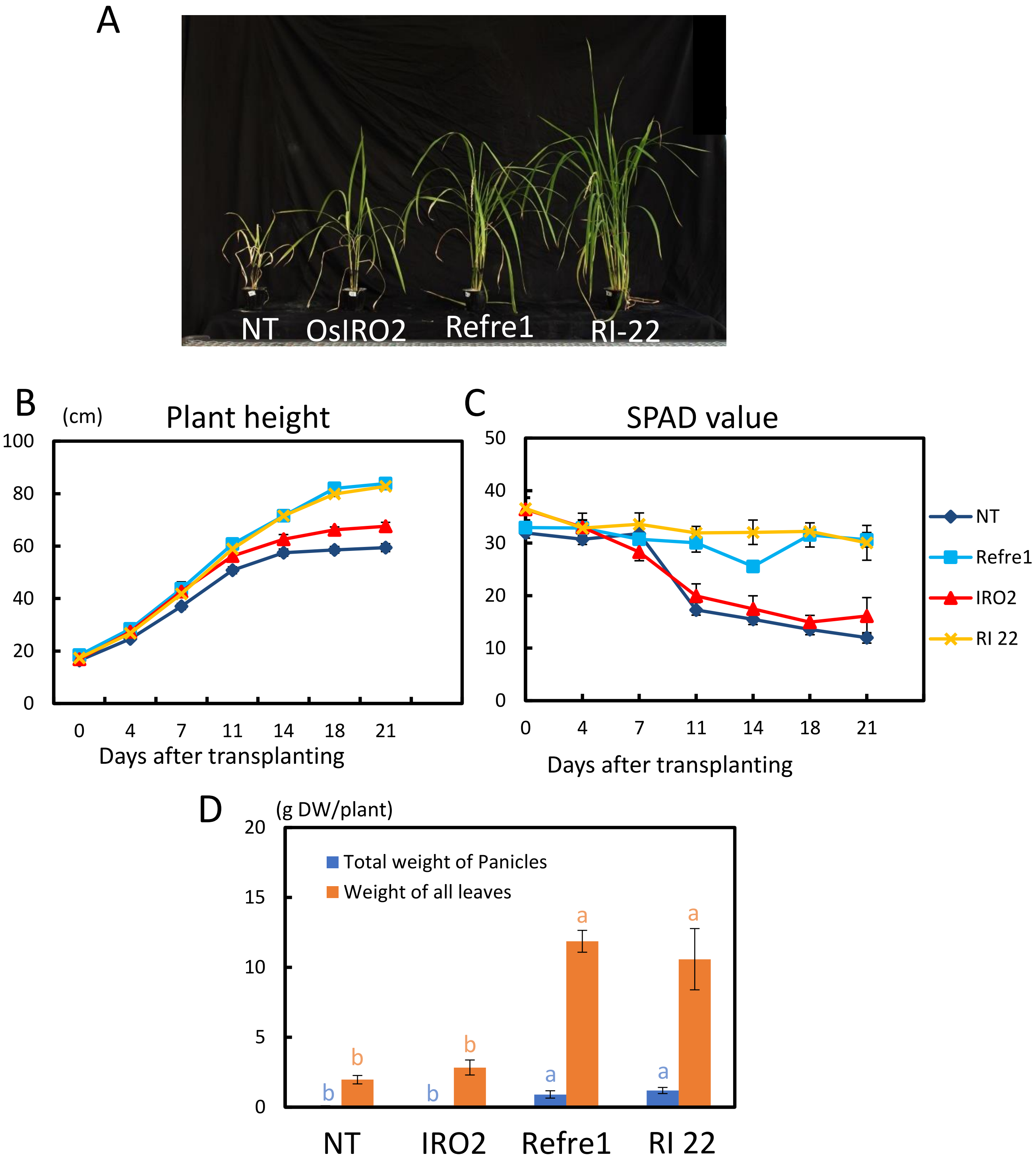

Supplementary Figure 6 | Cultivation condition 5: Growth test of RI rice on calcareous soil treated with ELT fertilizer (NPK plus micronutrients) under submerged condition. (A) Plant appearance at 84 days after transplanting. (B) Plant height. (C) SPAD value of the newest leaves. (D) Dry weight of panicles and dry weight of shoot after harvested. Five plants of each line were planted in different pots and data is the average of 5 plants (n=5). The water level was maintained continuously over 2 cm of the pot height. NT: non- transgenic lines, Refre1: the line with *OsIRT1* promoter-*refre1/372*, OsIRO2: the line with *35S* promoter-*OsIRO2*, RI-22: RI line number 22 with *OsIRT1* promoter-*refre1/372* and *35S* promoter-*OsIRO2.* Error bars represent ± 1 SE of biological replicates, n = 5.

Supplementary Figure 6

A

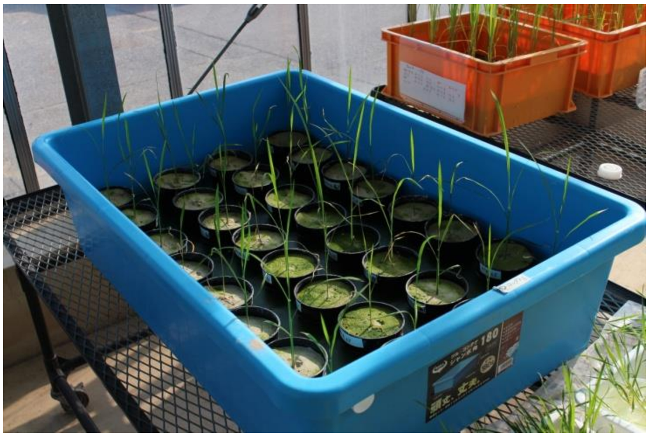

B

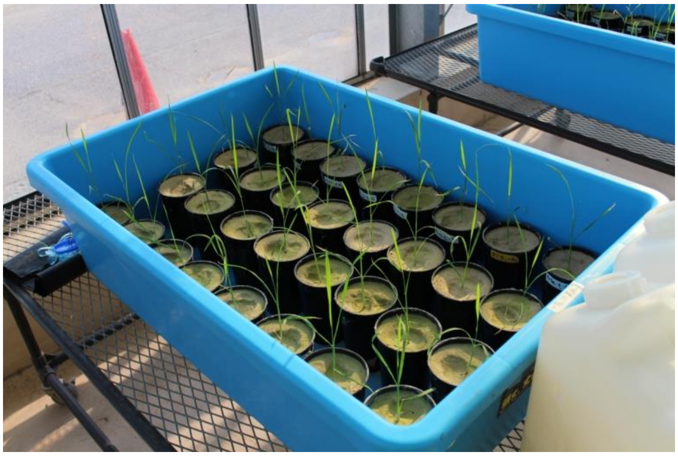

Supplementary Figure 7 | Growth conditions of T_3 IRI rice plants. (A) Main growth test 1 with ELT fertilizer and water submerged condition. (B) Main growth test 2 with Fe-deficient hydroponic solution and non-submerged condition.

Supplementary Figure 7

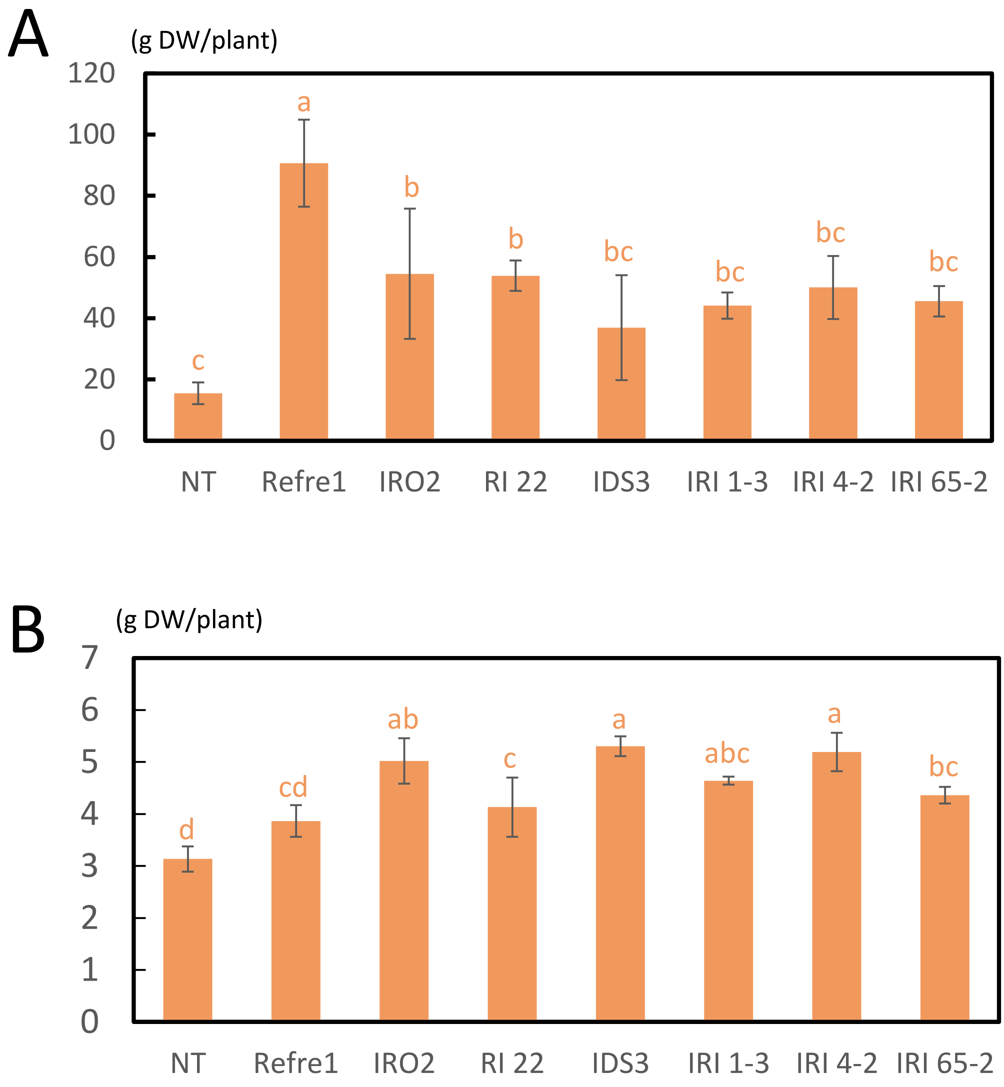

Supplementary Figure 8 | Biomass or dry weight of shoot of IRI lines compared to NT and other tested lines on calcareous soil under various water conditions. (A) Biomass of the plants harvested at 150 days after transplanting for submerged condition with Fe-sufficient fertilizer (Figure 4). (B) Biomass of the plants harvested at 105 days after transplanting for non-submerged condition with Fe-deficient fertilizer (Figure 5). NT: non- transgenic lines, Refre1: the line with *OsIRT1* promoter-*refre1/372*, OsIRO2: the line with *35S* promoter-*OsIRO2*, RI-22: RI line number 22 with *OsIRT1* promoter-*refre1/372* and *35S* promoter-*OsIRO2.* Error bars represent ± 1 SE of biological replicates, n = 4.

Supplementary Figure 8
